# Prevalence of Long COVID Symptoms Related to SARS-CoV-2 Strains

**DOI:** 10.3390/life13071558

**Published:** 2023-07-13

**Authors:** Teresita Aloè, Federica Novelli, Gianfranco Puppo, Valentina Pinelli, Emanuela Barisione, Elisa Trucco, Roberta Costanzo, Maria Grazia Covesnon, Federica Grillo, Patrizia Zoccali, Manlio Milanese, Sara Maniscalco, Elena Tagliabue, Ines Maria Grazia Piroddi, Simonetta Venturi, Maria Serra, Francesca Scordamaglia, Marta Ferrari, Antonella Serafini

**Affiliations:** 1 Interventional Pulmonology Unit, IRCCS Ospedale Policlinico San Martino, Largo Rosanna Benzi 10, 16100 Genoa, Italy; 2 Pulmonology Unit, ASL 5 Spezzino, 19124 La Spezia, Italy; 3 Pulmonology Unit, Ospedale Civile, 18100 Imperia, Italyantonella.serafini123@gmail.com (A.S.); 4 Pulmonology Unit, Ospedale Villa Scassi, ASL3 Genovese, 16100 Genoa, Italy; roberta.costanzo@asl3.liguria.it (R.C.);; 5 Anatomic Pathology Unit, IRCCS Ospedale Policlinico San Martino, 16100 Genoa, Italy; 6 Anatomic Pathology Unit, Università degli Studi di Genova, 16100 Genoa, Italy; 7 Pulmonology Unit, Ospedale S.Corona, 17027 Pietra Ligure, Italy; 8 Pulmonology Unit, IRCCS Ospedale Policlinico San Martino, 16100 Genoa, Italy

**Keywords:** Long COVID, variants, stains, post COVID, SARS-CoV-2

## Abstract

Background: Few studies have assessed the differences of patterns of Long COVID (L-COVID) with regards to the pathogenetic SARS-CoV-2 strains. Objectives: To investigate the relationship between demographic and clinical characteristics of acute phase of infection and the persistence of L-COVID symptoms and clinical presentation across different SARS-CoV-2 strains. Methods: In this observational-multicenter study we recorded all demographic and clinical characteristics, severity of infection, presence/persistence of symptoms of fatigue, dyspnoea and altered quality of life (QoL) at baseline and after 6 months, in a sample of Italian patients from Liguria between March 2020 and March 2022. Results: 308 patients (mean age 63.2 years; 55.5% men) with previous COVID were enrolled. Obese patients were 21.2% with a significant difference in obesity prevalence across the second and third wave (*p* = 0.012). Treatment strategies differed between waves (*p* < 0.001): more patients required invasive mechanical ventilation in the first wave, more patients were treated with high-flow nasal cannula/non-invasive ventilation in the in the second and more patients were treated with oxygen-therapy in the fourth wave. At baseline, a high proportion of patients were symptomatic (dyspnoea and fatigue), with impairment in some QoL indicators. A higher prevalence of patients with pain, were seen in the first wave compared to later infections (*p* = 0.01). At follow-up, we observed improvement of dyspnoea, fatigue and some dimensions of QoL scale evaluation such as mobility, usual activities, pain evaluations; instead there was no improvement in remaining QoL scale indicators (usual care and anxiety-depression). Conclusions: There were no significant differences in the prevalence of the most frequent L-COVID symptoms, except for QoL pain domain that was especially associated with classical variant. Our results show substantial impact on social and professional life and usual care activities. These findings highlight the importance of multidisciplinary post COVID follow-up care including mental health support and rehabilitation program.

## 1. Introduction

The term “Long COVID” (L-COVID) refers to the pattern of symptoms developed by a significant number of patients in the months following SARS-CoV-2 infection, regardless of the course of the acute phase or the levels of proinflammatory cytokines. These symptoms may be present constantly or have a remitting-recurrent course [1], and the most common include: chronic fatigue, myalgias, depression, sleep disturbances, dyspnoea, palpitations and chest pain [2]. This syndrome has brought many patients to specialist consultation after the apparent laboratory and radiological resolution of infection.

In particular, fatigue, either physical or mental [3], is one of the most frequent among the aforementioned symptoms, affecting quality of life [4]. It refers to a feeling of lack of energy that interferes with daily chores, and often follows other viral infections such as EBV, Influenza, SARS and MERS, with greater prevalence in the female gender and in patients with past history of anxiety or depression.

The steady evolution of SARS-CoV-2 virus through genome mutations has resulted in a new focus on L-COVID symptoms related to the SARS-CoV-2 variant involved. During the pandemic, new mutations in SARS-CoV-2 virus genome occurred in the replication stages and new variants emerged from the ancestral classical strain (identified in China in November 2019) divided into ‘variants of concern’ (VOC), ‘variants of interest’ (VOI) or ‘variants under monitoring’ (VUM) [5,6]. VOCs include Alpha (B.1.1.7), Beta (B.1.351), Gamma (P.1), Delta (B.1.617.2), and Omicron (B.1.1.529), which are associated with increased transmissibility and virulence and showed a wave-like trend in the incidence of SARS-CoV-2 cases with their outbreak and spread. However, very few papers have been published so far on the identification of descriptors of the acute COVID-19 phase that may significantly impact on post disease manifestations across different variants [7,8,9].

In this multi-centre observational study, promoted by the Italian Thoracic Society (ITS-AIPO), we systematically assessed demographic and clinical characteristics, severity of infection, the presence and persistence of symptoms of fatigue, dyspnoea, and altered quality of life correlated with the clinical presentation of L-COVID in a sample of patients, in the Ligurian region of Italy, who contracted COVID-19 infection from March 2020 to March 2022, divided according to SARS-CoV-2 waves.

## 2. Materials and Methods 

### 2.1. Patients, Study Design and Clinical Procedures

From November 2020 to July 2022, we enrolled 308 Ligurian patients in this multi-centric, observational and longitudinal study. Inclusion criteria were previous symptomatic infection from SARS-CoV-2 diagnosed by a positive SARS-CoV-2 swab test in the previous 18 months, and age > 18 years. Exclusion criteria were impossibility of the patient to read and sign the informed consent necessary for participation. The study protocol was approved by the Local Ethics Committee and all patients gave their explicit consent to data use.

All patients had a baseline visit, within 18 months from SARS-CoV-2 infection, and a follow-up visit 6 months later. During the baseline visit, we collected clinical and demographic data including smoking habit and BMI, comorbidity, severity of COVID-19, dyspnea, fatigue and quality of life.

Comorbidities were recorded by the list of diseases used to calculate the Age-Adjusted Charlson Comorbidity Index; we considered Charlson Score of 0–1 as absence of comorbidity, 2–3 as low comorbidity, 4–5 as high comorbidity and ≥6 as very high comorbidity [10].

COVID-19 severity was classified according to need for hospitalization and supplemental oxygen or mechanical ventilation, with a score ranging from 1 to 4: 1, not hospitalized; 2, hospitalized, not requiring supplemental oxygen or requiring supplemental traditional oxygen; 3, hospitalized, requiring High-flow nasal cannula (HFNC)/noninvasive ventilation (NIV); 4, hospitalized, requiring Intermittent Mandatory Ventilation (IMV) [11]. 

Dyspnoea was assessed using the modified Medical Research Council (MRC) dyspnoea scale [12].

Fatigue was evaluated and measured by the questionnaire Fatigue Assessment Scale (FAS) [13]. FAS is a 10-item self-reporting fatigue questionnaire; five questions reflect physical fatigue and 5 questions concern mental fatigue. The reliability and validity of the FAS are good generally as well as in L-COVID [14]. The response scale is a 5-point scale, with total scores ranging from 10 to 50, and high scores indicate greater fatigue. A total FAS score <22 corresponds to no fatigue, a score ≥22 identifies fatigue, a score ≥35 identifies extreme fatigue. 

Quality of Life was evaluated by the EQ-5D-3L questionnaire, a self-reporting instrument that measures five dimensions: mobility, self-care, usual activities, pain/discomfort and anxiety/depression. Each dimension has three levels: no problems, some problems and extreme problems [15].

**Figure 1:**
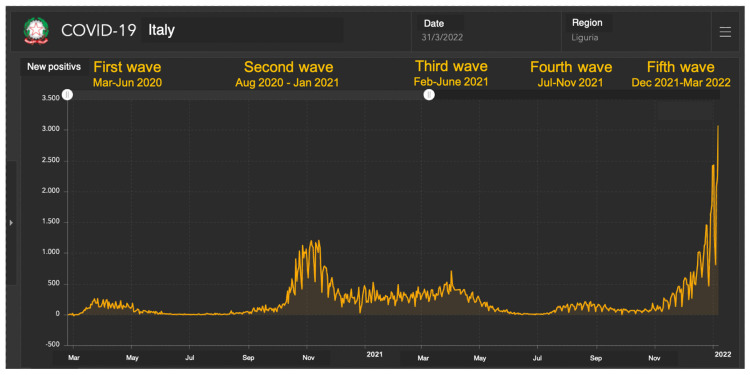
COVID-19 cases in Liguria from March 2020 and March 2022 (data from Department of Protezione Civile). Data from: https://mappe.protezionecivile.gov.it/it/mappe-e-dashboards-emergenze/dashboards-coronavirus/situazione-desktop/ accessed on 9 July 2023.

The subdivision of the pandemic in waves was based on the national database regarding the evolution of the COVID-19 pandemic, as daily reported by the Protezione Civile. This database collects the number of notified infections, COVID-19-related hospitalizations and deaths and recovered subjects by region [16]. For the type of variant in all waves we used the data of Hygiene Unit, San Martino Polyclinc Hospital, Genoa, Italy. In short: first wave from March to July 2020 (classical strain); second wave from August 2020 to January 2021 (classical strain-Alpha variant); third wave from February to June 2021 (Alpha, Beta, Gamma, Delta variants); fourth wave from July to November 2021 (Delta variant); fifth wave from December 2021 to March 2022 (Omicron variant) (Figure 1).

### 2.2. Statistics

Continuous normally distributed variables are expressed as mean ± standard error of the mean, while non-normally distributed data are expressed by median and interquartile range. Baseline characteristics were compared between the waves using ANOVA for age and Pearson chi-squared tests for categorical variables. Scores of dyspnoea, fatigue and quality of life were utilized as categorical variables. The comparison of paired categorical data was performed with McNemar or McNemar-Bowker test. *p* value < 0.05 was considered significant for all analyses. All analyses were conducted using IBM^©^ SPSS^©^ statistic software (version 21).

## 3. Results

### 3.1. Patients

From November 2020 to July 2022, we enrolled 308 patients who had fallen ill from COVID-19 disease from March 2020 to March 2022 and the median time from infection to visit was 153 days.

Table 1 describes baseline characteristics of the patients, subdivided by waves of COVID-19 in Liguria, Italy. Patients from the fifth wave were not included in the statistical analysis because of the small sample size. Mean age of patients was 63.2 years, 55.5% were men. In term of education level, 73.3% were within the High school level, with minimal differences between waves. Most of the patients were ex or non-smokers. A high percentage of patients were obese (21.2%) and, comparing the waves, we observed a significant difference in obesity prevalence, with more obese subjects in the second wave and less obese patients in the third wave (*p* = 0.012). Most patients had few or no comorbidities by AACCSI score, regardless of the waves they belonged to. Concerning disease severity, in the first wave the majority of patients had required IMV, in the second wave patients were mostly treated with HFNC/NIV, whereas in the fourth wave patients were treated principally with traditional oxygen (*p* < 0.001). In our sample only 13 patients (4.3%) had received COVID-19 vaccination, of which 7 (54%) in the fifth wave not included in the statistical analysis. 

### 3.2. Prevalence of Dyspnoea, Fatigue and Reduction in Quality of Life Identified on First and Second Visits

**Figure 2:**
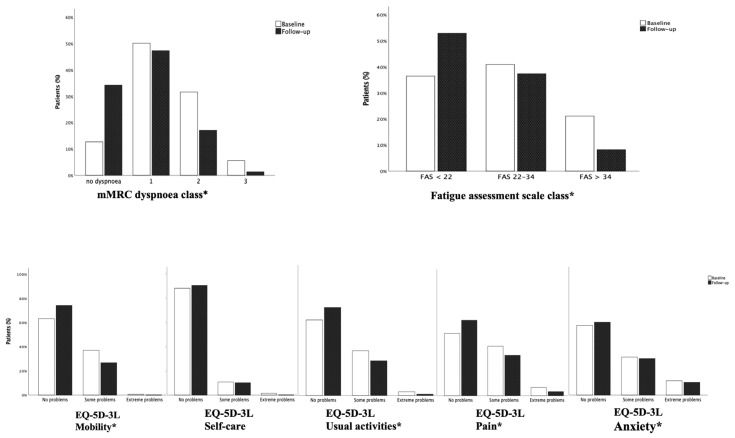
Differences in prevalence of dyspnea, fatigue and problems in quality of life at baseline and follow-up visit. The degree of dyspnoea (4 point scale), the degree of fatigue (3 point scale) and the degree of problems on 5 domains of quality of life (3 point scale) were compared at baseline and follow-up visit in the same subjects. The McNemar Bowker test was used for comparisons. * *p* < 0.05.

Median time from disease to baseline visit was 153.5 days (85.5–226.5). At that time, patients showed important persistence of symptoms: 269 patients (87.3%) still had dyspnoea, of whom 114 (42.2%) showing 2–3 grade MRC scale dyspnoea. One hundred and ninety-four patients (63%) complained of fatigue, described as extreme in 21.4% of cases. As for quality of life, 115 patients (37.3%) had some problems with mobility, 38 (12.3%) problems with self-care, 120 (38.9%) difficulty in undertaking usual activities, 148 (48%) lamented pain and 132 (42.8%) expressed feelings of anxiety or depression (Figure 2). 

At follow-up visit, carried out after a median time of 180 days (178–188), dyspnoea and fatigue improved, although a high percentage of patients were still symptomatic: 65.8% still had dyspnoea and 46.3% complained of fatigue. With reference to quality of life, we observed an improvement in mobility, usual activities and pain (26.5% had problems with mobility, 29% with usual activities and 37% with pain) but a substantially unchanged percentage of patients with problems regarding self-care (10.1%) and anxiety/depression (40.3%) (Figure 2).

### 3.3. Prevalence of Dyspnea, Fatigue and Reduction in Quality of Life at the First and Second Visit Compared by Waves of COVID-19

For this comparison, we excluded the fifth wave due to the small number of subjects. The time between infection and first interview was significantly different between waves (212 days (185–279) for the first, 169 (117–272) for the second, 136 (54–193) for the third and 105 (86–119) for the fourth; *p* < 0.001). At baseline visit, we did not observe any significant difference in the prevalence of dyspnoea or fatigue in patients infected in the different waves. As for quality of life, we found a higher prevalence of pain among patients infected in the first wave than in the later ones (first wave 75%, second wave 50.4%, third 42.1%, forth 37.5%, *p* = 0.01).

**Figure 3:**
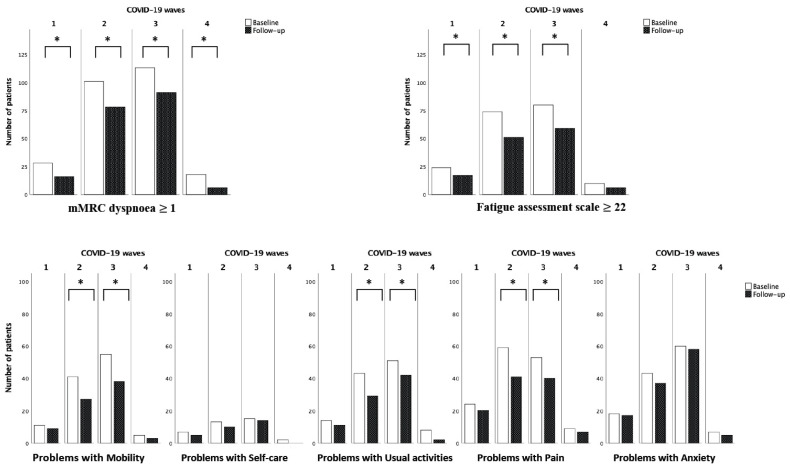
Differences in prevalence of dyspnoea, fatigue ed problems in quality of life at baseline and follow-up visit in the COVID-19 waves. Dyspnoea (Yes vs. No), fatigue (Yes vs. No) and the 5 domains of quality of life (Problems vs. no problems) were compared at baseline and follow-up visit in the same subjects. The McNemar test was used for comparisons. * *p* < 0.05.

Comparing the follow-up visits with baseline, prevalence of dyspnoea decreased significantly in all the waves, while fatigue decreased only in the 1st, 2nd and 3rd waves. Problems in mobility, usual activities and pain also showed a decrease in prevalence in the 2nd and 3rd waves (Figure 3).

## 4. Discussion/Conclusions

In this study, we aimed to investigate the persistence and prevalence of L-COVID-19 symptoms, such as dyspnea, fatigue and the impairment of quality of life, in a sample of patients from all over the Liguria region of Italy (population of approximately 1.5 milion), infected with different SARS-CoV-2 strains. Another purpose was to investigate the correlation between L-COVID syndrome and the differences in demographic data (age, smoking habit, BMI) and severity of COVID-19 disease seen in the different waves.

After the spread of the classical variant in December 2019, five new variants of concern have been widely reported, labelled as Alpha, Beta, Gamma, Delta and Omicron variants [4]. Although numerous studies have evaluated clinical outcomes related to acute phase COVID-19 in patients infected with different SARS-CoV-2 variants, to our knowledge, only a few studies have investigated the impact of different variants on L-COVID-19 syndrome symptoms [7,8,9,17].

Recent systematic reviews and meta-analyses confirmed that a high percentage (31–69%) of COVID–19 patients have experienced a range of long-term effects after three months from acute disease. The most commonly reported symptoms include fatigue, dyspnoea, cognitive impairment, anxiety, palpitations, muscle pain and arthralgia [18,19]. Similarly, in our cohort a high proportion of patients were still symptomatic after the acute phase. Regarding dyspnoea, 87% of patients were symptomatic at baseline and 65.8% still had dyspnea at follow-up with a median time of 326 days post-acute COVID. With regard to fatigue 63% of patients were symptomatic at baseline and 46.3% still complained of fatigue at follow-up. In other meta-analyses persistent fatigue and dyspnea were reported in more than half of L-COVID patients, even though these percentages vary greatly across different studies [18,19,20,21]. The exact mechanism responsible for long term complications of COVID-19 remains unknown, but a multi-factorial origin is probable. Pathological consequences of COVID infection, such as fibrotic remodeling after lung injury [22,23,24], endothelial cell dysfunction and pulmonary vasculature damage, abnormalities in the central and autonomous nervous system, metabolic abnormalities [20], uncontrolled immune response caused by coronavirus [25] and muscle mitochondrial dysfunction, may be the cause of the previously discussed symptoms, rather than psychological or environmental factors. 

Our study has not highlighted significant differences in the prevalence and persistence of dyspnoea and fatigue among different waves. Our data agree with the previous report by Morioka et al. [26], who did not find significant differences in the prevalence of each post COVID-19 symptom in the omicron infected patient group compared to other variant infected groups. Another study [27] found no differences in fatigue but more persistence of dyspnoea in individuals infected with the classical variant compared to those infected with Alpha or Delta variants. 

As for quality of life domains, at baseline visit, we found a higher prevalence of pain among patients infected in the first wave than in the later ones. The underlying pathophysiology of persistent symptoms could result from the release of large amounts of inflammatory cytokines and reactive oxygen species. A higher number of onset symptoms during the acute phase have been shown to be independent predictors for persistent symptoms, probably due to a persistent inflammatory state, at multivariable-analysis [25]. Patients infected with the classical variant showed a greater number of onset symptoms at hospitalization [27], this could explain the higher prevalence of post COVID symptoms (such as dyspnoea and pain) in patients infected with classical strains. 

We also found a reduction of mobility, usual activities and pain from baseline visit to follow up visit in all the waves (albeit not significantly in the 1st and 4th waves for the small number of patients), but no change in self-care and anxiety. In particular, one of the most deteriorated indicators is the persistence of anxiety/depression disorder. The overall prevalence of anxiety symptoms in our patients was 42.8% at baseline visit and 40.3% at follow-up visit. This result was higher than Premraj et al. [28] but coherent with Sykes et al. [29] who reported a prevalence of 47.8%. Notably, in the months preceding the pandemic, the Italian prevalence rate for anxiety and depression was below 17% (ISTAT, 2018). A previous study reported that SARS-CoV-2 infected survivors had higher rates of mental health consequences compared to the un-infected [30], highlighting a direct role of the infection besides other stress effects of the pandemic. The documented persistence of SARS-CoV-2 RNA in brain tissue or long term neuroinflammation [31] could be the link between COVID infection and cognitive and psychiatric manifestations of long COVID included anxiety symptoms. Our results agree with several reports suggesting that both anxiety and depression disorders persist longer than three months in COVID-19 survivors, indeed anxiety is reported between 10.4 to 35% of patients at one year from infection [32,33,34].

Furthermore, other risk factors have been associated with a higher probability of developing neuropsychiatric manifestations such as severity of COVID-19 acute phase (especially ICU admission), female sex, presence of comorbidities and a history of mental health diseases [35,36]. Moreover, fatigue, one of the most prevalent long-term symptoms, decreases quality if life and may interfere with psychological disorders and daily activities [36].

Notably, 10% of patients in our cohort complained of persistent impairment in usual self-care, resulting in considerable chronic morbidity and rising costs for health services.

Another purpose of our study was to investigate differences in demographic data (age, smoking habit, BMI) and severity of COVID-19 between the successive waves. In our cohort, we observed a higher prevalence of obesity in patients infected during the second wave when classical and Alfa were the prevalent variants in our region. There are conflicting data regarding the prevalence of obesity in the different waves [26,27,37] in the literature. It is possible that our results could be partly related to the influence of the lockdown period (March–May 2020), which was particularly stringent in Italy, on activity levels and obesity [38,39]. In this contest, it is now well established that obesity and other cardiometabolic risk factors promote inflammation and endothelial dysfunction [40]. Currently, there is no clear reason why patients with certain comorbidities could be infected with one variant and not another.

Another finding of this study was the progressive reduction in severity of COVID-19 disease from the first to the last wave. In our cohort, patients infected during the first wave required more IMV, conversely more patients in the second wave required more HFNC/NI; in the fourth wave, patients were predominantly treated with traditional oxygen. It is worth highlighting that the adoption of IMV significantly changed in frequency as waves followed waves, thanks to improved understanding of COVID-19 pathophysiology [41]. This could have partly affected severity comparison between subsequent waves, especially for the first wave. In agreement with our results, previous studies by Challen et al. [42], Davies et al. [43], Grint et al. [44], reported greater hospitalization and worse outcome in patients infected by Alpha variant.

The major strength of this report is the study design which was multi-centre and focuses on the subgroup analysis between SARS-CoV-2 waves/variants; furthermore, the majority of our cohort was unvaccinated at the time of the infection, which makes this variable non-confounding, even though a small cohort of vaccinated patients was present, preventing us from assessing the possible role of vaccination on the long COVID outcome in different SARS-CoV-2 waves/variants. L-COVID symptoms have been evaluated via standardized and validated set of questionnaires and this is a major advantage, even though they do not include less frequent symptoms which would require specific, in depth analysis. Our study has some other limitations; firstly, our retrospective cohort displays a relevant proportion of missing values regarding laboratory data, since molecular virus identification was not available. Hence, SARS-CoV-2 variants were presumed by epidemiological data based on prevalent circulating viral strains in our region in the specific period. Secondly, the sample size was not large enough to provide better statistical power. Lastly, our study has been carried out on patients who were mostly hospitalized and this may have caused a selection bias (especially with regard to psychological aspects). Thus, our clinical observation needs to be confirmed by larger controlled prospective studies. 

In conclusion, this real-world study investigated the prevalence of post COVID-19 symptoms in patients infected by different SARS-CoV-2 waves. Due to small study cohort, we did not observe statistically significant differences in the prevalence of the most common symptoms such as dyspnoea, fatigue and quality of life impairment, except for the pain indicator that is more frequently associated with the classical variant. 

Furthermore, while severity and outcome of the acute phase depend on the infectivity, virulence and pathogenicity of different SARS-CoV-2 strains, our data suggests that each viral variant considered could trigger long COVID syndrome through the persistence of the inflammatory response.

Our results provide additional evidence concerning long term COVID-19 sequalae, showing a substantial deleterious impact on social and professional life, and usual care activities. These findings highlight the importance of planning post COVID-19 follow up care with a multidisciplinary approach, including mental health support, and rehabilitation programs.

## Figures and Tables

**Table 1:** Demographic data, smoking habit, obesity, comorbidity and severity of COVID-19 of patients, divided by waves of COVID-19.

	All *n* = 308	1st Wave *n* = 32	2nd Wave *n* = 117	3rd Wave *n* = 126	4th Wave *n* = 24	5th Wave *n* = 9	*p*-Value ^a^
Age (mean ± SD)	63.2 ± 11.8	62.5 ±12.7	63.9 ± 10.9	63.6 ± 12.1	60.6 ± 11.9	58.5 ± 15.7	0.618
Gender							
Men	171 (55.5%)	16 (50%)	66 (56.4%)	73 (57.9%)	14 (58.3%)	2 (22.2%)	0.876
Women	137 (44.5%)	16 (50%)	51 (43.6%)	53 (42.1%)	10 (41.7%)	7 (77.8%)	
Hospital of evaluation							
San Martino H, Genova	131 (42.6%)	32 (100%)	53 (45.3%)	26 (20.6%)	20 (83.3%)	0	<0.001
ASL 5, La Spezia	61 (19.8%)	0	18 (15.4%)	32 (25.4%)	2 (8.3%)	9 (100%)	
Civile H, Imperia	56 (18.2%)	0	24 (20.5%)	31 (24.6%)	1 (4.2%)	0	
Villa Scassi H, Genova	50 (16.2%)	0	18 (15.4%)	32 (25.4%)	0	0	
Santa Corona H, Pietra Ligure	10 (3.2%)	0	4 (3.4%)	5 (4%)	1 (4.2%)	0	
Title of study							
Primary and Secondary school	98 (31.8%)	7 (21.9%)	40 (34.2%)	42 (33.3%)	7 (29.2%)	2 (22.2%)	0.043
High school	128 (41.5%)	8 (25%)	44 (37.6%)	62 (49.2%)	8 (33.3%)	6 (66.7%)	
Laurea	43 (13.9%)	8 (25%)	14 (12%)	16 (12.7%)	4 (16.7%)	1 (11.1%)	
No title	15 (4.8%)	4 (12.5%)	5 (4.3%)	3 (2.4%)	3 (12.5%)	0	
Unknown	24 (7.9%)	5 (15.6%)	14 (12%)	3 (2.4%)	2 (8.3%)	0	
Cigarette smoking (293)							
Never smoker	165 (56.3%)	15 (46.9%)	63 (57.3%)	72 (60%)	10 (45.5%)	5 (55.6%)	0.622
Former smoker	119 (40.6%)	16 (50%)	42 (38.2%)	46 (38.3%)	11 (50%)	4 (44.4%)	
Current smoker	9 (3.1%)	1 (3.1%)	5 (4.5%)	2 (1.7%)	1 (4.5%)	0	
Obesity (*n* = 293)							
Not obese	231 (78.8%)	27 (84.4%)	77 (69.4%)	103 (86.6%)	17 (73.9%)	7 (87.5%)	0.012
Obese	62 (21.2%)	5 (15.6%)	34 (30.6%)	16 (13.4%)	6 (26.1%)	1 (12.5%)	
AACCI Score							
0–1	126 (40.9%)	13 (40.6%)	59 (50.4%)	41 (32.5%)	10 (41.7%)	3 (33.3%)	0.269
2–3	116 (37.7%)	13 (40.6%)	38 (32.4%)	52 (41.3%)	9 (37.5%)	4 (44.4%)	
4–5	50 (16.2%)	6 (18.8%)	14 (12%)	25 (19.8%)	3 (12.5%)	2 (22.2%)	
≥6	16 (5.2%)	0	6 (5.1%)	8 (6.3%)	2 (8.3%)	0	
Severity of disease							
Not requiring hospitalization	26 (8.4%)	1 (3.1%)	11 (9.4%)	9 (7.1%)	1 (4.2%)	4 (44.4%)	<0.001
Admitted to hospital, not requiring oxygen or requiring traditional oxygen	103 (33.4%)	15 (46.9%)	25 (21.4%)	47 (37.3%)	15 (62.5%)	1 (11.1%)	
Admitted to hospital, requiring HFNC or CPAP/NIV	155 (50.3%)	9 (28.1%)	76 (65.0%)	60 (47.6%)	6 (25%)	4 (44.4%)	
Admitted to hospital, requiring IMV	24 (7.8%)	7 (21.9%)	5 (4.3%)	10 (7.9%)	2 (8.3%)	0	

AACCI: Age-Adjusted Charlson Comorbidity Index. ^a^ 5th wave excluded from statystical analysis for small number of subjects; comparisons between the 4 waves by ANOVA for age and chi square for categorical variables. H—hospital; HFNC—High-flow nasal cannula; CPAP/NIV—Continuous Positive Airway Pressure/non-invasive ventilation: IMV—Intermittent Mandatory Ventilation.

## Data Availability

The data that support the finding of this study are available on request from the corresponding author.
